# Vitamin D as a Key Mediator Between C-reactive Protein to Albumin Ratio and Congestive Heart Failure in an Elderly Population: An Innovative Exploration Using the NHANES Database

**DOI:** 10.31083/RCM37740

**Published:** 2025-07-11

**Authors:** Yufeng Wei, Zhaofeng Zhang

**Affiliations:** ^1^Department of Pharmacy, Ganzhou People’s Hospital, 341000 Ganzhou, Jiangxi, China; ^2^Department of Pharmacology, Ganzhou Dermatosis Hospital, 341000 Ganzhou, Jiangxi, China

**Keywords:** vitamin D, C-reactive protein to albumin ratio (CAR), congestive heart failure (CHF), mediation effect analysis, National Health and Nutrition Examination Survey (NHANES), cross-sectional study

## Abstract

**Background:**

The C-reactive protein-to-albumin ratio (CAR), a marker of inflammation and nutritional status (calculated as C-reactive protein [CRP]/albumin [ALB]), is associated with increased mortality in congestive heart failure (CHF). However, whether vitamin D modulates the CAR-CHF relationship remains unclear. Using data from the National Health and Nutrition Examination Survey (NHANES), this study aimed to investigate the mediating role of vitamin D in the association between CAR and CHF among older adults, with implications for cardiovascular disease prevention.

**Methods:**

Data from NHANES 2001–2010 were analyzed, including adults aged ≥65 years. Multivariate logistic regression was used to assess the independent association of CAR and 25-hydroxyvitamin D [25(OH)D] with CHF. Pearson correlation evaluated bivariate relationships between continuous variables (vitamin D, CAR), while Spearman correlation assessed associations between the dichotomous CHF status and continuous variables (vitamin D, CAR). Mediation analysis (Hayes’ PROCESS Model 4, 5000 bootstrap samples) tested whether 25(OH)D mediated the CAR-CHF link. Subgroup analyses explored effect modification by age, sex, and comorbidities.

**Results:**

A total of 4128 participants (mean age: 70.0 years; 55.81% male) were included, with 247 (5.98%) diagnosed with CHF. Vitamin D deficiency (25(OH)D <20 ng/mL) and insufficiency (20–30 ng/mL) were prevalent (71.2%). Key findings included: Bivariate associations: Lower 25(OH)D correlated with higher CAR (r = –0.12, *p* = 0.004) and increased CHF risk (Spearman ρ = –0.061, *p* < 0.01), while CAR was positively correlated with CHF (Spearman ρ = 0.080, *p* < 0.01). Multivariate analysis: CAR was an independent risk factor for CHF (adjusted OR for highest vs. lowest quartile: 1.96, 95% confidence interval (CI): 1.31–2.95, *p* < 0.001; *p*-trend < 0.001. Vitamin D sufficiency (25(OH)D ≥30 ng/mL) was associated with a lower CHF risk compared to deficiency (25(OH)D <20 ng/mL, OR: 0.56, 95% CI: 0.38–0.83, *p* = 0.003), indicating that deficiency was indirectly linked to higher risk. Mediation effect: 25(OH)D partially mediated the CAR-CHF association, explaining 3.00% of the total effect (indirect effect: 0.002, 95% CI: 0.001–0.005, *p* = 0.039). Predictive value: CAR had modest accuracy for CHF (area under the curve (AUC) = 0.597, 95% CI: 0.560–0.634), with an optimal cut-off of 0.149 (sensitivity: 59.1%, specificity: 56.4%).

**Conclusion:**

Elevated CAR and vitamin D deficiency are independently associated with increased CHF risk in older adults. Vitamin D partially mediated the association between CAR and CHF, underscoring its role in linking inflammation/nutrition status to cardiovascular risk. Clinicians should monitor both biomarkers in CHF prevention, prioritizing inflammation control and vitamin D repletion in high-risk populations.

## 1. Introduction

Congestive heart failure (CHF) is a common cardiovascular disease characterized by symptoms such as dyspnea, malaise (usually manifesting as reduced exercise tolerance) and fluid retention (e.g., peripheral oedema), as well as elevated plasma natriuretic peptide levels [1]. With the rapid aging of the world’s population, CHF has become a growing public health concern, placing a significant burden on the health status and quality of life of the elderly [2]. This condition, characterized by progressive cardiac dysfunction and impaired hemodynamics, highlights the urgent need for improved diagnostic strategies and therapeutic interventions to mitigate its impact on vulnerable aging populations [3].

Traditional risk factors for CHF, including hypertension, smoking, diabetes mellitus, genetic predisposition and obesity, are well established [4, 5, 6]. However, in addition to these well-recognized traditional risk factors, emerging novel risk factors are being identified, which may hold significant prognostic and therapeutic implications. Despite substantial progress in the treatment of CHF, early diagnosis and the identification of factors capable of accurately predicting the risk of developing CHF remain critical for early disease prevention and necessitate further in-depth research.

Inflammatory processes are implicated in every stage of CHF onset, progression, and complication [7, 8]. C-reactive protein (CRP), a well-known archetypal marker of inflammation, has been positively correlated with the risk of CHF. Meanwhile, albumin levels are indicative of the body’s nutritional status, and a decrease in albumin is associated with a poor prognosis in CHF patients. In recent years, the CRP to albumin ratio (CAR), as a novel inflammatory indicator, has garnered significant attention. Studies have shown that elevated CAR levels are closely associated with the development of various chronic diseases [8, 9], particularly cardiovascular diseases [10]. CAR can comprehensively reflect the body’s inflammatory and nutritional status, and changes in its level may indicate the level of risk of cardiovascular diseases, especially CHF [11, 12, 13].

Beyond its established importance in calcium and phosphorus metabolism and bone health, researchers worldwide have conducted numerous investigations into the relationships among vitamin D, CAR, and cardiovascular diseases. An accumulating body of evidence suggests that vitamin D has an effect on the cardiovascular system [14, 15, 16, 17]. Similarly, elevated CAR levels have been recognized as an independent risk factor for cardiovascular diseases [18, 19]. Elevated CAR levels signify the body’s chronic inflammatory state, and inflammatory responses are known to play a pivotal role in the development of cardiovascular diseases. These responses can trigger pathological processes such as atherosclerosis and thrombosis, which are crucial in the progression of cardiovascular disorders. Conducting an in-depth exploration of the role of vitamin D in the relationship between CAR and CHF among the elderly population is highly promising. Such a study could simultaneously help elucidate the underlying pathogenesis of CHF and also provide a solid theoretical basis for formulating effective preventive and therapeutic strategies. This research direction may open up new avenues for understanding the complex mechanisms underlying CHF and contribute to the development of more targeted and efficient interventions for this prevalent disease among the elderly.

Notwithstanding the existing research, the mediating role of vitamin D in the association between CAR and CHF has received relatively scant attention, and the conclusions drawn from previous studies have been inconsistent. Therefore, the present study, which utilizes the nationally representative National Health and Nutrition Examination Survey (NHANES) database, is of great theoretical and practical significance. It aims to comprehensively explore the intricate relationships among vitamin D, CAR, and CHF, potentially shedding new light on the underlying pathological mechanisms of CHF, hopefully contributing to advancing methods of prevention and treatment for this prevalent condition among the elderly population.

## 2. Methods

### 2.1 Study Design and Subject Inclusion and Exclusion

The NHANES is a nationally representative continuous health survey project conducted by the National Center for Health Statistics (NCHS) under the Centers for Disease Control and Prevention (CDC). The database covers a wide range of information on demographics, physical measurements, laboratory tests, and health questionnaires, providing a rich data resource for medical research. No additional informed consent or ethical review was required as all study participants provided informed consent and the NCHS Institutional Review Board approved the study. All methods were performed in accordance with relevant guidelines and regulations.

**Fig. 1.**
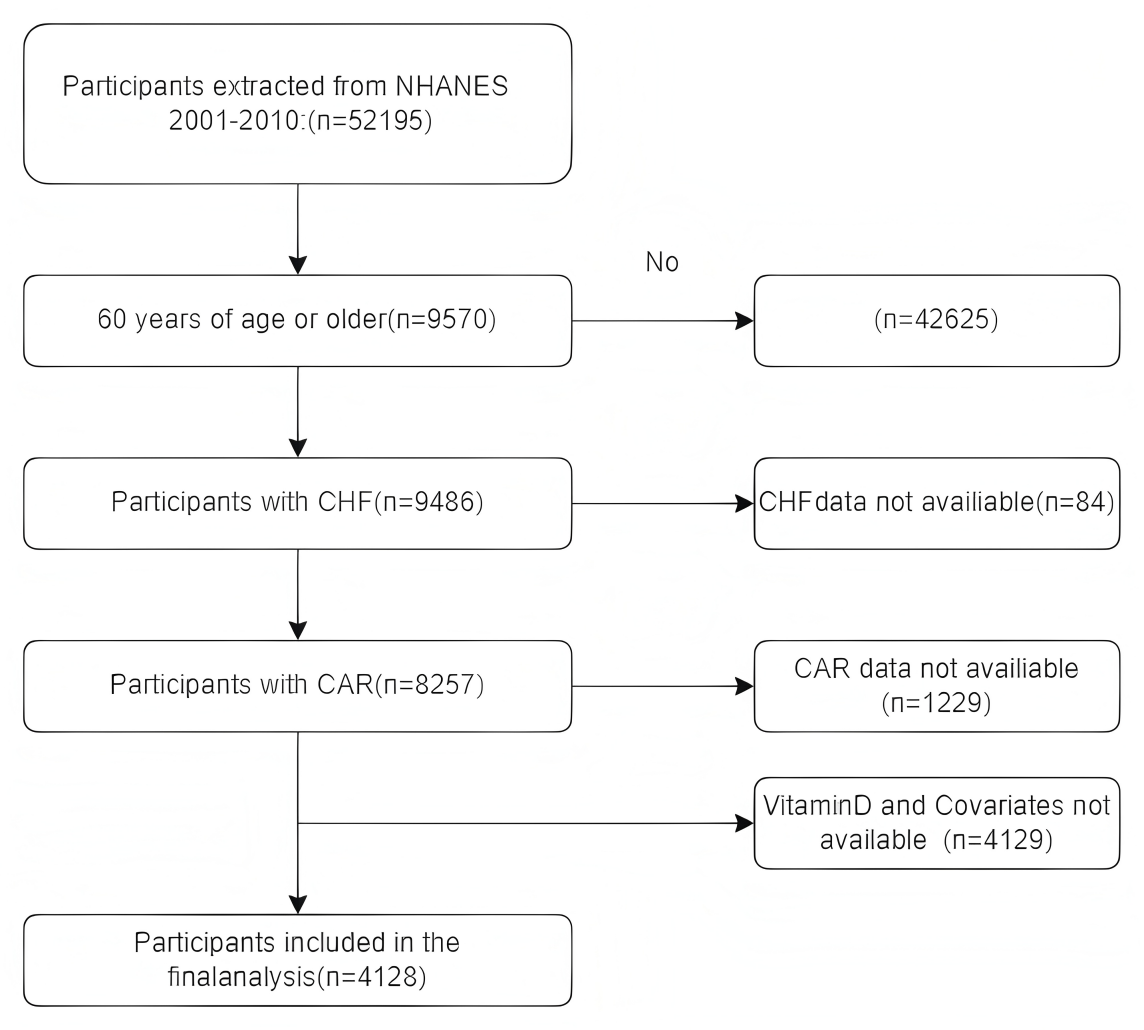
**Flow diagram of selecting populations for analysis**. CHF, congestive heart failure; CAR, C-reactive protein to albumin ratio; NHANES, National Health and Nutrition Examination Survey.

In this study, data from the NHANES database spanning the years 2001–2010 were carefully screened to identify the elderly population aged 60 years or older. The inclusion criteria were designed to ensure the availability of complete data regarding vitamin D levels, the CAR, and disease-related markers of CHF. Conversely, individuals with severe hepatic and renal disorders, malignancies, autoimmune diseases (specifically rheumatoid arthritis), and other conditions that could potentially impact vitamin D metabolism and the inflammatory response were excluded. Following this rigorous screening procedure, a total of 4128 elderly individuals were selected for inclusion in the study. The sample selection process is presented in Fig. 1 through a detailed flowchart, which demonstrates the steps and criteria employed to determine the final study sample.

Flowchart illustrating the process of selecting study participants aged 60 years and above from the NHANES database between 2001 and 2010, including inclusion and exclusion criteria, and the final inclusion of 4128 individuals.

### 2.2 Serum Vitamin D Concentration

Serum samples from NHANES 2001–2010 were evaluated by liquid chromatography-tandem mass spectrometry. Serum total vitamin D levels (nmol/L) were calculated by totaling the levels of 25-hydroxyvitamin D2 and 25-hydroxyvitamin D3. Vitamin D deficiency was defined as 25(OH)D <20 ng/mL, vitamin D insufficiency was defined as 20 ng/mL ≤ 25(OH)D < 30 ng/mL, and vitamin D sufficiency was defined as 25(OH)D ≥30 ng/mL [20]. Participants with missing data on serum vitamin D concentration were removed from this study.

### 2.3 Definition of CHF

CHF disease history was obtained through an interview. Participants were asked, ‘Has a doctor or other health professional ever told you that you have congestive heart failure?’ Patients were considered to have CHF if they answered ‘yes’ to the above question [21].

### 2.4 Definition of CAR

CAR was defined as serum CRP (mg/dL)/serum albumin (g/dL) and both CRP and albumin (ALB) were collected at a mobile screening center and sent to the laboratory for analysis. Prior to collection, blood samples were screened against specific exclusion criteria [9, 22].

### 2.5 Ascertainment of Covariates

In our study, several potential covariates were considered, including demographic factors, living status variables, disease status indicators, and biochemical indices. Demographic information encompassed sex, age, race, marital status, and educational attainment. Living status variables involved smoking habits, alcohol consumption patterns, and body mass index (BMI). Disease status included hypertension, diabetes mellitus, cancer, hepatic and renal diseases, and autoimmune diseases (specifically rheumatoid arthritis). The information regarding hypertension, diabetes mellitus, cancer, hepatic and renal diseases, and rheumatoid arthritis was obtained through questionnaires. In terms of biochemical indices, total cholesterol (TC) and aspartame nontransferable (AST) were utilized. All data collection and handling processes adhered to a standardized protocol to systematically investigate the potential impact of these covariates on the relationship between the CAR and CHF.

### 2.6 Statistical Analyses

CAR was categorized into quartiles (Q1 ≤0.0262, Q2 0.0262–0.055, Q3 0.055–0.1167, Q4 >0.1167) to evaluate dose-response relationships, with Q1 (lowest CAR) as the reference group. Continuous variables were tested for normality using the Shapiro-Wilk test; non-normally distributed data were reported as median (IQR). Categorical variables were presented as frequencies (%). Group comparison: Analysis of Variance (ANOVA) with Bonferroni correction compared normally distributed variables across CAR quartiles; Kruskal-Wallis test was used for non-normally distributed variables across multiple groups, with Mann-Whitney U tests for post-hoc pairwise comparisons when significant group differences were found. Correlation & regression: Pearson’s correlation analysis explored the relationship between vitamin D (25(OH)D, ng/mL), CAR, and the relationship between CHF and vitamin D, and CAR using spearman analysis. Multivariate logistic regression models assessed independent effects of CAR and vitamin D (treated as a continuous variable) on CHF, with stepwise adjustment: Model 1: Unadjusted; Model 2: Adjusted for gender, age, race; Model 3: Model 2 + education, alcohol, smoking, BMI; Model 4: Model 3 + hypertension, diabetes, TC. Mediation & ROC: Hayes’ PROCESS Model 4 (adjusted for Model 4 covariates) tested the mediating role of vitamin D, using 5000 bootstrap samples for 95% confidence intervals (CIs) of indirect effects. Receiver operating characteristic (ROC) curves with 1000-bootstrap resampling evaluated CAR’s predictive accuracy for CHF, calculating the area under the curve (AUC) and identifying the optimal cut-off via Youden’s index (sensitivity + specificity –1). Subgroup analysis: Likelihood ratio tests evaluated effect modification by demographic (age, sex, race) and clinical (hypertension, diabetes) factors to assess interaction effects. All analyses were performed using SPSS 27.0 (IBM Corp., Armonk, NY, USA), with statistical significance set at *p* < 0.05.

## 3. Results

### 3.1 Clinical Baseline Features of the Subjects

Table 1 presents a summary of the baseline characteristics of the study population, stratified by the presence or absence of CHF. Among the included elderly adults, 28.78% had vitamin D deficiency, 42.44% had vitamin D insufficiency, and 28.78% had sufficient vitamin D levels. Further analysis indicated that the rates of vitamin D deficiency and insufficiency were higher in men compared to women. Racial disparities were also observed, with non-Hispanic whites having the highest prevalence of vitamin D deficiency. Out of the 4128 participants, 247 were diagnosed with CHF, resulting in an overall CHF prevalence of 5.98% in the study population. The mean age of the participants was 70 years. Regarding gender distribution, 55.81% of the participants were male, and 44.19% were female. Statistical analyses revealed that men, individuals with higher educational attainment, drinkers, smokers, those with hypertension, and patients with vitamin D deficiency had a significantly higher risk of developing CHF compared to their counterparts (all *p* < 0.05). Notably, within the CHF group, although non-drinkers accounted for a higher proportion (72.87%) compared to drinkers (27.13%), this does not contradict the observed higher risk of CHF in drinkers when compared across groups (CHF vs. No-CHF). This within-group proportion highlights the complexity of analyzing risk factors and underscores the importance of considering both within- and between-group perspectives in interpreting such data.

**Table 1.** **Baseline characteristics of the CHF group versus the non-CHF**.

Characteristics		No-CHF (n = 3881)	CHF (n = 247)	*p*
Age (years)		69.0 (63.0, 76.0)	72.0 (66.0, 80.0)	<0.001
BMI (kg/m^2^)		28.0 (24.8, 31.5)	29.5 (26.4, 35.4)	<0.001
ALB (g/dL)		4.2 (4.0, 4.4)	4.1 (3.8, 4.2)	<0.001
AST (U/L)		24.0 (20.0, 28.0)	23.0 (19.0, 27.0)	<0.005
TC (mg/dL)		201.0 (173.0, 230.0)	180.0 (147.0, 215.0)	<0.001
CRP (mg/dL)		0.2 (0.1, 0.5)	0.3 (0.1, 0.8)	0.002
CAR (mg/g)		0.054 (0.0, 0.1)	0.077 (0.0, 0.2)	0.002
Vitamin D (ng/mL)		25.3 (19.5, 31.3)	23.0 (16.0, 28.6)	<0.001
Gender, n (%)				<0.001
	Male	2135 (55.01)	169 (68.42)	
	Female	1746 (44.99)	78 (31.58)	
Race, n (%)				0.122
	Mexican American	692 (17.83)	30 (12.15)	
	Others	296 (7.63)	19 (7.69)	
	Non-Hispanic White	2239 (57.69)	149 (60.32)	
	Non-Hispanic Black	654 (16.85)	49 (19.84)	
Education, n (%)				0.002
	High school graduate	1321 (34.04)	108 (43.72)	
	Some college or above	2560 (65.96)	139 (56.28)	
Marital status, n (%)				0.043
	Have a partner	2419 (62.33)	138 (55.87)	
	No partner	1462 (37.67)	109 (44.13)	
Drinker, n (%)				<0.001
	Yes	618 (15.92)	67 (27.13)	
	No	3263 (84.08)	180 (72.87)	
Smoker, n (%)				<0.001
	Yes	2303 (59.34)	173 (70.04)	
	No	1578 (40.66)	74 (29.96)	
Hypertension, n (%)				<0.001
	Yes	2100 (54.11)	185 (74.90)	
	No	1781 (45.89)	62 (25.10)	
Diabetes, n (%)				<0.001
	Yes	661 (17.03)	103 (41.70)	
	No	3220 (82.97)	144 (58.30)	
Vitamin D, n (%)				0.003
	25(OH)D <20 ng/mL	1097 (28.30)	91 (36.84)	
	20 ng/mL ≤ 25(OH)D < 30 ng/mL	1647 (42.40)	105 (42.51)	
	25(OH)D ≥30 ng/mL	1137 (29.30)	51 (20.65)	

Notes: Continuous and categorical variables were displayed individually as mean ± SD or proportions. Abbreviations: BMI, body mass index; ALB, albumin; AST, aspartate aminotransferase; TC, total cholesterol; CRP, C-reactive protein; CHF, congestive heart failure; CAR, C-reactive protein to albumin ratio.

Clinical baseline characteristics of the study population stratified by the presence or absence of CHF. This includes the distribution of vitamin D levels (deficiency, insufficiency, sufficiency), CHF prevalence, age, gender distribution, and the risk differences of CHF among various demographic and clinical characteristics (e.g., higher risk of CHF in males, higher education, drinkers, smokers, hypertensive patients, and vitamin D deficient individuals).

Table 2 displays the CAR values of the participants stratified into quartiles. The median CAR value was 0.055. Q1 represents values below 0.0262, Q2 encompasses values ranging from 0.0262 to 0.055, Q3 includes values from 0.055 to 0.1167, and Q4 corresponds to values above 0.1167. The level of CAR was found to be correlated with multiple factors, including age, gender, BMI, smoking status, and alcohol consumption. Specifically, there was a statistically significant association between CAR levels and age (*p* = 0.035), but the trend was not a simple increase with age. Men exhibited higher CAR levels compared to women. A positive correlation was detected between BMI and CAR levels, such that the higher the BMI, the higher the CAR level. Smokers showed a progressive increase in proportion across higher CAR quartiles (*p* < 0.001), suggesting smokers were associated with elevated CAR levels. Additionally, drinking status distribution differed significantly across CAR quartiles (*p* = 0.008), indicating a potential association. Moreover, the prevalence of CHF increased in tandem with the increasing CAR values of the participants. The CHF prevalence rates for Q1, Q2, Q3, and Q4 were 4.03%, 4.85%, 6.41%, and 8.66% respectively, with a statistically significant difference (*p* < 0.001).

**Table 2.** **Baseline characteristics of the study population based on CAR**.

Characteristics		Q1 (n = 1041)	Q2 (n = 1030)	Q3 (n = 1029)	Q4 (n = 1028)	*p*
Age (years)		70.0 (64.0, 76.0)	69.0 (63.0, 76.0)	70.0 (63.0, 76.0)	68.0 (63.0, 75.0)	0.035
BMI (kg/m^2^)		26.3 (23.3, 29.1)	27.7 (25.0, 31.0)	28.8 (25.8, 32.2)	30.1 (26.0, 34.5)	<0.001
ALB (g/dL)		4.3 (4.1, 4.5)	4.2 (4.1, 4.4)	4.2 (4.0, 4.4)	4.0 (3.8, 4.2)	<0.001
AST (U/L)		24.0 (21.0, 28.0)	24.0 (21.0, 28.0)	23.0 (20.0, 28.0)	22.0 (19.0, 26.0)	<0.001
TC (mg/dL)		196.0 (169.0, 224.0)	200.0 (172.0, 232.0)	202.0 (174.0, 232.5)	201.0 (172.0, 230.8)	0.005
Vitamin D (ng/mL)		26.4 (20.7, 32.3)	25.4 (20.0, 31.1)	24.6 (18.6, 30.1)	23.8 (16.8, 30.1)	<0.001
Gender, n (%)						<0.001
	Male	636 (61.10)	594 (57.67)	579 (56.27)	495 (48.15)	
	Female	405 (38.90)	436 (42.33)	450 (43.73)	533 (51.85)	
Race, n (%)						<0.001
	Mexican American	167 (16.04)	175 (16.99)	204 (19.83)	176 (17.12)	
	Others	97 (9.32)	75 (7.28)	67 (6.51)	76 (7.39)	
	Non-Hispanic White	638 (61.29)	618 (60.00)	594 (57.73)	538 (52.33)	
	Non-Hispanic Black	139 (13.35)	162 (15.73)	164 (15.94)	238 (23.15)	
Education, n (%)						<0.001
	High school graduate	302 (29.01)	380 (36.89)	347 (33.72)	400 (38.91)	
	Some college or above	739 (70.99)	650 (63.11)	682 (66.28)	628 (61.09)	
Marital status, n (%)						<0.001
	Have a partner	699 (67.15)	649 (63.01)	644 (62.59)	565 (54.96)	
	No partner	342 (32.85)	381 (36.99)	385 (37.41)	463 (45.04)	
Drinker, n (%)						0.008
	Yes	137 (13.16)	182 (17.67)	185 (17.98)	181 (17.61)	
	No	904 (86.84)	848 (82.33)	844 (82.02)	847 (82.39)	
Smoker, n (%)						<0.001
	Yes	571 (54.85)	606 (58.83)	642 (62.39)	657 (63.91)	
	No	470 (45.15)	424 (41.17)	387 (37.61)	371 (36.09)	
Hypertension, n (%)						<0.001
	Yes	532 (51.10)	545 (52.91)	580 (56.37)	628 (61.09)	
	No	509 (48.90)	485 (47.09)	449 (43.63)	400 (38.91)	
Diabetes, n (%)						0.154
	Yes	184 (17.68)	182 (17.67)	183 (17.78)	215 (20.91)	
	No	857 (82.32)	848 (82.33)	846 (82.22)	813 (79.09)	
CHF, n (%)						<0.001
	No	999 (95.97)	980 (95.15)	963 (93.59)	939 (91.34)	
	Yes	42 (4.03)	50 (4.85)	66 (6.41)	89 (8.66)	

Notes: Continuous and categorical variables were displayed individually as mean ± SD or proportions.

### 3.2 Logistic Regression Analyses

Pearson correlation analysis showed that vitamin D level was significantly negatively correlated with CAR (r = –0.12, *p* = 0.004), i.e., the lower the level of vitamin D, the higher the level of CAR; Given that CHF is a dichotomous variable, a Spearman correlation analysis was performed. The results showed a significant negative correlation between CHF and vitamin D (ρ = –0.061, *p* < 0.01), implying that higher levels of vitamin D were associated with a lower risk of developing CHF. On the contrary, there was a significant positive correlation between CHF and CAR (ρ = 0.080, *p* < 0.01), suggesting that higher levels of CAR are associated with an increased likelihood of CHF. This suggests that vitamin D may influence CAR levels by modulating the inflammatory response, which in turn may have an impact on the development of CHF.

The associations between CAR and CHF, as well as vitamin D and CHF, are presented in Table 3, derived from multivariate logistic regression analyses. In the unadjusted model, CAR was significantly and positively associated with CHF (odds ratio (OR) = 2.09, 95% CI: 1.49–2.94, *p* < 0.001). This association persisted after adjusting for gender, age, and race in Model 2 (OR = 2.10, 95% CI: 1.49–2.96, *p* < 0.001). Model 3, which additionally adjusted for education, alcohol consumption, smoking, and BMI, also showed a significant association (OR = 2.05, 95% CI: 1.45–2.88, *p* < 0.001). Even after further adjusting for hypertension, diabetes, and TC in Model 4, the significant correlation remained (OR = 2.16, 95% CI: 1.51–3.09, *p* < 0.001). These results collectively demonstrate a robust positive correlation between CAR and CHF across all models. Meanwhile, the negative correlation between vitamin D and CHF, along with corresponding OR, CI, and *p*-values, is also detailed in Table 3.

**Table 3.** **Odds ratios and 95% confidence intervals for CHF according to CAR and vitamin D**.

Variables		Model 1		Model 2		Model 3		Model 4	
		OR (95% CI)	*p*	OR (95% CI)	*p*	OR (95% CI)	*p*	OR (95% CI)	*p*
CAR (continuous)		2.09 (1.49~2.94)	<0.001	2.10 (1.49~2.96)	<0.001	2.05 (1.45~2.88)	<0.001	2.16 (1.51~3.09)	<0.001
CAR (quartile)									
	Quartile 1	Reference		Reference		Reference		Reference	
	Quartile 2	1.21 (0.80~1.85)	0.366	1.24 (0.81~1.89)	0.315	1.01 (0.65~1.54)	0.981	1.11 (0.72~1.71)	0.644
	Quartile 3	1.63 (1.10~2.42)	0.016	1.69 (1.13~2.51)	0.01	1.28 (0.85~1.93)	0.235	1.50 (0.99~2.27)	0.057
	Quartile 4	2.25 (1.55~3.29)	<0.001	2.50 (1.71~3.67)	<0.001	1.72 (1.16~2.57)	0.008	1.96 (1.31~2.95)	0.001
	Vitamin D (continuous)	0.97 (0.96~0.98)	<0.001	0.96 (0.95~0.98)	<0.001	0.97 (0.96~0.99)	<0.001	0.97 (0.95~0.99)	<0.001
Vitamin D (groups)									
	25(OH)D <20 ng/mL	Reference		Reference		Reference		Reference	
	20 ng/mL ≤ 25(OH)D < 30 ng/mL	0.77 (0.57~1.03)	0.076	0.69 (0.51~0.94)	0.018	0.76 (0.55~1.03)	0.081	0.76 (0.55~1.05)	0.095
	25(OH)D ≥30 ng/mL	0.54 (0.38~0.77)	<0.001	0.48 (0.33~0.71)	<0.001	0.58 (0.39~0.85)	0.005	0.56 (0.38~0.83)	0.003

Notes: Model 1: No adjustment for covariates. Model 2: Adjust: Gender, Age, Race. Model 3: Adjust: Gender, Age, Race, Drinking, Smoking, BMI. Model 4: Adjust: Gender, Age, Race, Drinking, Smoking, BMI, Hypertension, Diabetes, TC. Abbreviations: OR, odds ratio; 95% CI, 95% confidence interval.

After stratifying CAR into quartiles, the strong correlation between CAR and CHF was still evident, and the trend test was statistically significant (*p* < 0.05). In Model 4, comparing the highest quartile of CAR with the lowest quartile revealed a 96% increase in the incidence of CHF (OR = 1.96, 95% CI: 1.31–2.95, *p* < 0.001). Moreover, a significant negative correlation was found between vitamin D and CHF. After adjusting for the variables in Model 4, each unit increase in the serum concentration of vitamin D was associated with a 0.97-fold reduction in the odds of developing CHF (OR = 0.97; 95% CI: 0.95–0.99; *p* < 0.001). In Model 1, for the category of 20 ng/mL ≤ 25(OH)D < 30 ng/mL, the statistical software output shows an OR of 0.77, with a 95% CI of 0.57∼1.03, and a *p*-value of 0.076.

These findings suggest that both vitamin D and CAR are independent risk factors for CHF. This further corroborates the crucial role of vitamin D and CAR in the pathogenesis of CHF, and demonstrates that their effects on CHF remain significant even after controlling for other common risk factors.

The results of the linear regression analyses exploring the relationship between the CAR and vitamin D are presented in Table 4. In the unadjusted model, CAR was negatively associated with vitamin D levels (β = –1.68; 95% CI: –2.82– –0.54; *p* = 0.004), indicating that as CAR increased, vitamin D levels tended to decrease. However, after adjusting for variables such as gender, age, race, education, drinking status, smoking, BMI, hypertension, diabetes, and TC, this significant association was no longer observed.

**Table 4.** **Multiple linear regression analysis of the association between CAR and vitamin D**.

Variables		Model 1		Model 2		Model 3		Model 4	
		β (95% CI)	*p*	β (95% CI)	*p*	β (95% CI)	*p*	β (95% CI)	*p*
CAR (continuous)		–1.68 (–2.82~–0.54)	0.004	–1.13 (–2.19~–0.07)	0.037	–0.65 (–1.71~0.41)	0.228	–0.70 (–1.75~0.36)	0.196
CAR (quartile)									
	Quartile 1	Reference		Reference		Reference		Reference	
	Quartile 2	–1.42 (–2.20~–0.63)	<0.001	–1.25 (–1.98~–0.52)	<0.001	–0.79 (–1.52~–0.06)	0.035	–0.80 (–1.53~–0.06)	0.033
	Quartile 3	–1.99 (–2.77~–1.20)	<0.001	–1.68 (–2.41~–0.95)	<0.001	–1.00 (–1.74~–0.25)	0.008	–1.03 (–1.77~–0.28)	0.007
	Quartile 4	–2.79 (–3.57~–2.00)	<0.001	–2.01 (–2.75~–1.27)	<0.001	–1.02 (–1.79~–0.26)	0.008	–1.06 (–1.82~–0.29)	0.007

Notes: Model 1: No adjustment for covariates. Model 2: Adjust: Gender, Age, Race. Model 3: Adjust: Gender, Age, Race, Drinking, Smoking, BMI. Model 4: Adjust: Gender, Age, Race, Drinking, Smoking, BMI, Hypertension, Diabetes, TC.

### 3.3 ROC Analysis

**Fig. 2.**
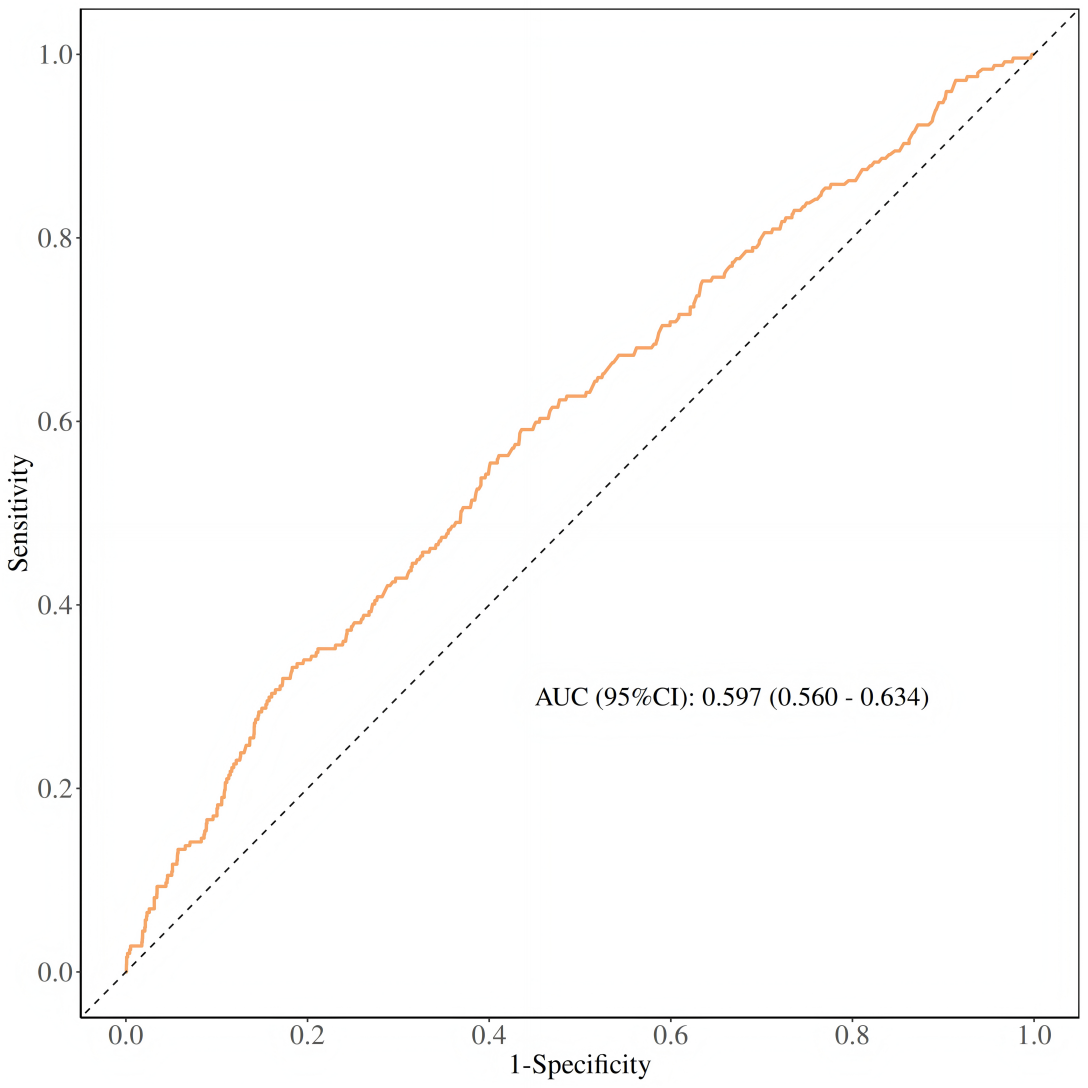
**ROC curves and the AUC values of CAR in diagnosing CHF**. AUC, area under the curve; ROC, receiver operating characteristic.

To accurately evaluate the predictive power of the CAR level for CHF, we calculated the AUC of the ROC curve for the study subjects. The results are presented in Fig. 2. Our study findings revealed that the AUC value of the CAR level was 0.597. While this suggests CAR has a marginal predictive ability for CHF compared to random guessing (AUC = 0.5), the modest AUC indicates that CAR alone holds limited clinical predictive value for CHF. These results warrant cautious interpretation, and additional research is needed to explore biomarker combinations or refined models with enhanced predictive accuracy. Through further analysis, the optimal cut-off value of CAR was determined to be 0.149. At this cut-off value, the sensitivity for predicting CHF was 59.1% and the specificity was 56.4% (since specificity = 1 – 0.436), achieving an optimal balance between sensitivity and specificity for prediction.

### 3.4 Subgroup Analyses and Interaction Tests

To evaluate the stability of the association between the CAR and CHF and to identify potential differences among different subgroups, we conducted subgroup analyses. The results of these analyses are presented in Table 5. In the sensitivity test, we observed that the ‘*p*-value for interaction’ was greater than 0.05. This finding indicated that the positive association between CAR and CHF was generally stable and consistent across the overall population.

**Table 5.** **Subgroups analysis for the associations between CAR and CHF**.

Variables		n (%)	OR (95% CI)	*p*	*p* for interaction
All patients		4128 (100.00)	2.12 (1.50~3.00)	<0.001	
Gender					0.082
	Male	2304 (55.81)	2.71 (1.77~4.14)	<0.001	
	Female	1824 (44.19)	1.53 (0.90~2.59)	0.113	
Race					0.313
	Mexican American	722 (17.49)	3.37 (1.26~9.01)	0.016	
	Others	315 (7.63)	20.65 (1.40~304.80)	0.027	
	Non-Hispanic White	2388 (57.85)	2.13 (1.37~3.31)	<0.001	
	Non-Hispanic Black	703 (17.03)	1.30 (0.37~4.53)	0.680	
Education					0.314
	High school graduate	1429 (34.62)	2.75 (1.52~4.97)	<0.001	
	Some college or above	2699 (65.38)	1.88 (1.25~2.81)	0.002	
Drinker					0.357
	Yes	685 (16.59)	2.54 (1.44~4.49)	0.001	
	No	3443 (83.41)	1.91 (1.25~2.93)	0.003	
Smoker					0.187
	Yes	2476 (59.98)	1.96 (1.38~2.80)	<0.001	
	No	1652 (40.02)	4.98 (1.67~14.90)	0.004	
Hypertension					0.836
	Yes	2285 (55.35)	2.03 (1.34~3.07)	<0.001	
	No	1843 (44.65)	2.18 (1.14~4.17)	0.019	
Diabetes					0.108
	Yes	764 (18.51)	5.61 (1.50~21.00)	0.010	
	No	3364 (81.49)	1.91 (1.35~2.70)	<0.001	
Age					0.422
	60–70	2330 (56.44)	1.76 (0.89~3.47)	0.106	
	≥70	1798 (43.56)	2.31 (1.47~3.64)	<0.001	

### 3.5 Mediation of Vitamin D

**Fig. 3.**
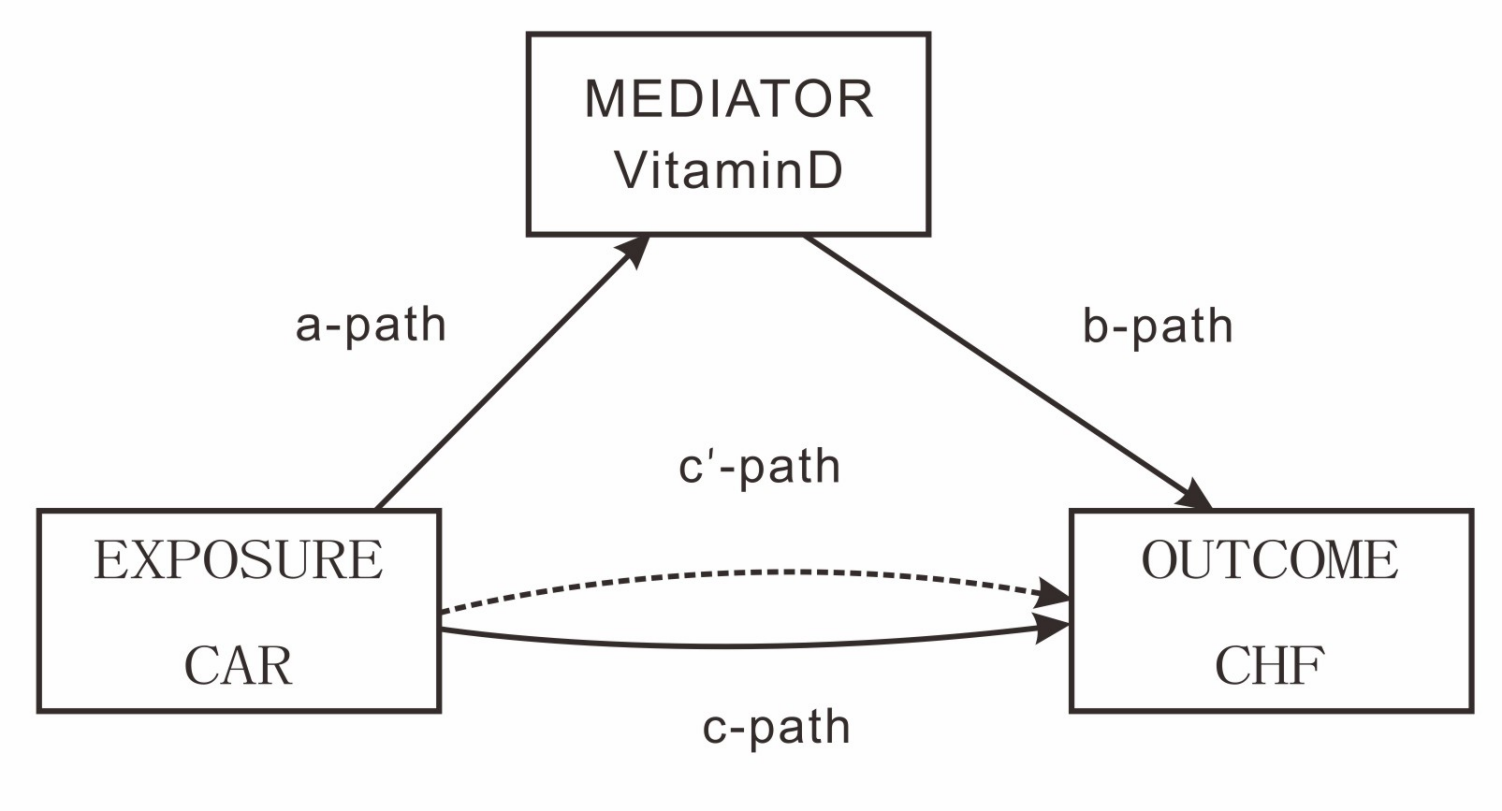
**Path diagram of the mediation analysis models**. In the mediation analysis framework, the “c - path” represents the total effect of EXPOSURE (CAR) on OUTCOME (CHF), while the “c’ - path” denotes the direct effect, excluding the influence mediated by vitamin D.

Mediation analyses were performed to assess whether vitamin D mediates the association between CAR and CHF disease occurrence. Mediation analyses were performed by establishing three pathways. For this purpose, we set up three pathways: (1) exposure to mediator; (2) mediator to outcome (direct effect); and (3) exposure to outcome (total effect). The total effect reflects the sum of the direct and mediated (indirect) effects. The percentage of mediated effects was calculated using the following formula: (mediated effect/total effect) × 100 [23]. Mediation analyses were performed to assess whether vitamin D mediated the association between CAR and CHF development. The model for mediation analysis is shown in Fig. 3 and the pathway for mediation analysis is shown in Table 6.

**Table 6.** **Mediation analysis for the associations between CAR and CHF**.

Independent variable mediator	Mediator	Total effect	Indirect effect	Direct effect	Proportion mediated
		Coefficient (95% CI)	Coefficient (95% CI)	Coefficient (95% CI)	
CAR	vitamin D	0.081 (0.052~0.111)	0.002 (0.001~0.005)	0.079 (0.05~0.108)	3.00%

Note: In mediation analyses, adjustments were made for Gender, Age, Race, Drinking, Smoking, AST, TC.

Mediation effect analysis models and results for the role of vitamin D in the association between CAR and CHF. This includes the total effect of CAR on CHF, the direct effect, the mediating effect of vitamin D, and the proportion of the total effect mediated by vitamin D (3.00%), indicating that vitamin D plays a partial mediating role in the relationship between CAR and CHF.

The results of the mediation effect analysis indicated that the total effect of the CAR on CHF was significant (β = 0.081, 95% CI: 0.052–0.111, *p* < 0.01). This demonstrates that the overall impact of CAR on congestive heart failure is very significant. When vitamin D was introduced as a mediator variable, the direct effect of CAR on CHF remained significant (β = 0.079, 95% CI: 0.05–0.108, *p* < 0.01), yet the effect value decreased, the *p*-value is much less than 0.05, indicating that CAR has a significant direct impact on CHF. The mediating effect of vitamin D was also significant (β = 0.002, 95% CI: 0.001–0.005, *p* = 0.039). Vitamin D partially mediated the association between CAR and CHF, but its contribution was low (3.00%).

This finding holds significant importance for clinical practice. Physicians can utilize this cut-off value to assess the risk of patients, enabling them to promptly identify individuals at potential risk of CHF and take appropriate preventive and therapeutic measures. For instance, if a patient’s CAR level exceeds this cut-off value, it indicates that the patient is at a high risk of developing CHF, and further in-depth examination and close monitoring of the patient are necessary.

## 4. Discussion

Vitamin D partially mediated the association between CAR and CHF, but its percentage contribution was low (3.00%), but the indirect effect was statistically significant (*p* = 0.039). Analyzing a sample of 4128 individuals aged 60 years and above, we identified a significant association between CAR and CHF. This finding implies that individuals with elevated CAR levels are more likely to develop CHF. Remarkably, this correlation remained strong even after adjusting for a comprehensive set of covariates in the fully adjusted model (Model 4). Subgroup analyses and sensitivity tests further indicated that the associations were relatively stable across different subgroups, with no significant differences observed. Overall, there was a positive association between CAR and the development of CHF. Simultaneously, vitamin D was found to partially mediate the relationship between CAR and CHF, emphasizing the importance of closely monitoring both the inflammatory levels (represented by CAR) and vitamin D levels. This also highlights the significance of improving the management of patients with low vitamin D levels for the prevention of cardiovascular diseases.

An in-depth exploration of the NHANES database has unveiled the critical role of vitamin D in mediating the relationship between CAR and CHF. CAR, as a novel biomarker, integrates information on inflammation and nutritional status of the organism, providing valuable insights into the mechanistic study of the disease and prognostic assessment of patients. This finding is consistent with previous findings by other scholars and further validates the potential of CAR in clinical applications [24, 25, 26]. Elevated CRP levels signify an augmented inflammatory response [27]. CRP concentration is a strong predictor of the occurrence or exacerbation of heart failure events in patients with heart failure and those at risk [11, 28]. While decreased ALB levels suggest poor nutritional status. Both serum CRP and ALB are important prognostic indicators of the risk of death in patients with CHF.

Our findings are consistent with numerous prior studies [15, 29, 30, 31], indicating that vitamin D deficiency is significantly associated with an increased risk of CHF. As a steroid hormone with a wide range of biological functions, vitamin D exerts complex and diverse effects within the cardiovascular system. On one hand, vitamin D can regulate the renin-angiotensin system (RAS) [32, 33]. By inhibiting the expression of the renin gene, it reduces the production of angiotensin Ⅱ, which ultimately leads to a decrease in blood pressure and a reduction in the pressure load on the vascular wall, thus protecting the cardiovascular system. On the other hand, vitamin D has anti-inflammatory and antioxidant properties [34, 35, 36]. It can suppress the activation of inflammatory cells and the release of inflammatory factors, alleviate oxidative stress damage, and preserve the integrity and function of vascular endothelial cells. These actions collectively contribute to reducing the risk of cardiovascular diseases. In conclusion, both vitamin D deficiency and elevated CAR levels are associated with an increased risk of CHF.

The results of the mediation effect analysis strongly confirm that vitamin D plays a significant mediating role in the relationship between CAR and CHF. This reveals that CAR not only has a direct impact on the occurrence of CHF but also indirectly influences it by modulating vitamin D levels. Specifically, elevated CAR levels may trigger an inflammatory response and disrupt vitamin D metabolism, resulting in decreased vitamin D levels. Vitamin D deficiency, in turn, further diminishes its protective effects on the cardiovascular system, thereby increasing the risk of CHF.

This finding underscores that, in the prevention and treatment of cardiovascular diseases, apart from focusing on the inflammatory and nutritional status reflected by CAR, it is essential to pay close attention to maintaining and regulating vitamin D levels. By doing so, healthcare providers can more effectively control the risk of CHF and potentially improve patient outcomes.

## 5. Strengths and Limitations of the Study

In comparison to previous research, the present study, leveraging a large sample from the NHANES database and applying more rigorous research methodologies and statistical analyses, has further affirmed the associations among vitamin D, CAR, and CHF. Notably, it has, for the first time, clarified the mediating role of vitamin D in the relationship between CAR and CHF. While several prior studies have recognized the association between vitamin D deficiency and CHF, they have not explored its mediating role in the connection between inflammatory markers and CHF. Therefore, this study fills this research gap and provides a novel perspective for a more in-depth understanding of the relationships among vitamin D, CAR, and CHF.

However, this study is not without limitations. First, the NHANES database adopts a cross-sectional study design, inherently restricting the establishment of causal relationships among vitamin D, CAR, and cardiovascular disease. Prospective studies are vital to confirm such causal links. Second, although certain potential confounders were accounted for, unmeasured confounders might still affect the findings. Third, supplementary analyses on the correlation between cardiovascular disease and medications/specific treatments in cardiovascular patients were lacking, which could impact the interpretation of investigated relationships and require further exploration. More importantly, the CHF disease history relied on interviews, potentially introducing recall bias. Additionally, the absence of objective verification via tests (e.g., echocardiography) poses a risk of misdiagnosis. These limitations in CHF diagnosis data collection should be acknowledged.

## 6. Conclusion

The present study demonstrated for the first time that vitamin D plays a key mediating role in the association between CAR and CHF by analyzing data from an elderly population (mediating effect share 3.00%, *p* = 0.039). Cross-sectional analyses showed that vitamin D deficiency (25(OH)D <20 ng/mL) and elevated CAR (OR = 2.16, 95% CI: 1.51–3.09) were independent risk factors for CHF. Mechanistically, vitamin D may block CAR-mediated pathophysiological processes by inhibiting the inflammatory response (lowering CRP) and improving endothelial function.

In clinical practice, combined monitoring of CAR and vitamin D levels may help to identify people at risk for CHF. Prompt supplementation of vitamin D-deficient patients, especially those with elevated CAR, may slow the progression of CHF by reducing the inflammatory load. However, this study was limited by a cross-sectional design, and causality needs to be further validated in a prospective cohort study. Future randomized controlled trials are needed to assess the synergistic effect of vitamin D supplementation combined with anti-inflammatory interventions in the prevention of CHF in order to improve the theoretical basis and guide clinical translation.

## Availability of Data and Materials

Detailed descriptions of NHANES, all data, and guidance on analytical approaches can be found at https://www.cdc.gov/nchs/nhanes/index.htm.
